# Fasting durations of Steller sea lion pups vary among subpopulations—evidence from two plasma metabolites

**DOI:** 10.1093/conphys/coad084

**Published:** 2023-11-24

**Authors:** Stephanie G Crawford, Robert H Coker, Todd M O’Hara, Greg A Breed, Tom Gelatt, Brian Fadely, Vladimir Burkanov, Patricia M Rivera, Lorrie D Rea

**Affiliations:** Department of Biology and Wildlife and Institute of Northern Engineering, University of Alaska Fairbanks, 1764 Tanana Loop, Fairbanks, Alaska 99775, USA; Montana Center for Work Physiology and Exercise Metabolism, University of Montana, 32 Campus Drive, Missoula, Montana 59812, USA; Veterinary Integrative Biosciences, School of Veterinary Medicine & Biomedical Sciences, Texas A&M University, 402 Raymond Stotzer Parkway, Bldg 2, College Station, Texas 77843, USA; Institute of Arctic Biology, University of Alaska Fairbanks, Fairbanks, Alaska 99775, USA; Marine Mammal Laboratory, Alaska Fisheries Science Center, National Marine Fisheries Service, National Oceanic and Atmospheric Administration, 7600 Sand Point Way N.E., Bldg. 4, Seattle, Washington 98115, USA; Marine Mammal Laboratory, Alaska Fisheries Science Center, National Marine Fisheries Service, National Oceanic and Atmospheric Administration, 7600 Sand Point Way N.E., Bldg. 4, Seattle, Washington 98115, USA; Marine Mammal Laboratory, Alaska Fisheries Science Center, National Marine Fisheries Service, National Oceanic and Atmospheric Administration, 7600 Sand Point Way N.E., Bldg. 4, Seattle, Washington 98115, USA; Center for Alaska Native Health Research, Institute of Arctic Biology, University of Alaska Fairbanks, 2141 Koyukuk Drive, Fairbanks, Alaska 99775, USA; Institute of Northern Engineering, University of Alaska Fairbanks, 1764 Tanana Loop, Fairbanks, Alaska 99775, USA

**Keywords:** fasting, metabolites, pinnipeds

## Abstract

Geographic differences in population growth trends are well-documented in Steller sea lions (*Eumetopias jubatus*), a species of North Pacific pinniped listed under the U.S. Endangered Species Act in 1990 following a marked decline in population abundance that began during the 1970s. As population growth is intrinsically linked to pup production and survival, examining factors related to pup physiological condition provides useful information to management authorities regarding potential drivers of regional differences. During dam foraging trips, pups predictably transition among three fasting phases, distinguished by the changes in the predominant metabolic byproduct. We used standardized ranges of two plasma metabolites (blood urea nitrogen and β–hydroxybutyrate) to assign pups to fasting categories (n = 1528, 1990–2016, 12 subpopulations): *Recently Fed–Phase I* (digestion/assimilation–expected hepatic/muscle glycogen usage), *Phase II* (expected lipid utilization), transitioning between *Phases II–III* (expected lipid utilization with increased protein reliance), or *Phase III* (expected protein catabolism). As anticipated, the majority of pups were classified as *Recently Fed–Phase I* (overall mean proportion = 0.72) and few pups as *Phase III* (overall mean proportion = 0.04). By further comparing pups in *Short* (*Recently Fed–Phase II*) and *Long* (all other pups) duration fasts, we identified three subpopulations with significantly (*P* < 0.03) greater proportions of pups dependent upon endogenous sources of energy for extended periods, during a life stage of somatic growth and development: the 1) central (0.27 ± 0.09) and 2) western (0.36 ± 0.13) Aleutian Island (declining population trend) and 3) southern Southeast Alaska (0.32 ± 0.06; increasing population trend) subpopulations had greater *Long* fast proportions than the eastern Aleutian Islands (0.10 ± 0.05; stabilized population). Due to contrasting population growth trends among these highlighted subpopulations over the past 50+ years, both density-independent and density-dependent factors likely influence the dam foraging trip duration, contributing to longer fasting durations for pups at some rookeries.

## Teaser

Steller sea lion pup fasting durations were longer (i.e., proportion of pups in Long fasts) in two subpopulations associated with continued population decline and a third with steady population growth (n=1528 pups, 12 subpopulations), suggesting both density-dependent and independent influences upon pup fasting duration.

## Introduction

### Background

As a fasting–adapted species, all age and sex classes of Steller sea lions (SSL; *Eumetopias jubatus*) enter fasting states as part of their annual cycle. Following parturition onshore during the late spring, SSL dams fast on the rookery during the perinatal period while provisioning neonatal pups (Pitcher *et al.*, 2001; Maniscalco *et al.*, 2006; Hastings and Jemison, 2016; Kuhn *et al.*, 2017; Maniscalco and Parker, 2018). After 1–2 weeks (Table 1) females depart to forage, leaving the newborn pup onshore to digest and assimilate the latest meal and likely begin the first fast, depending upon the duration of the dam’s foraging trip (Milette and Trites, 2003; Maniscalco *et al.*, 2006; Burkanov *et al.*, 2011). Within a day, the dam typically returns to nurse her newborn pup (Table 1). This alternation between foraging and fasting, for both the dam and her pup, continues for approximately 1 year, and sometimes longer, with some observations of dams concurrently nursing a yearling and newborn (Pitcher and Calkins, 1981; Maniscalco *et al.*, 2006). These elements of the maternal attendance pattern—the perinatal nursing period, foraging trip duration and shore visit duration—influence a pup’s growth and development, body condition and subsequent fasting capabilities. To successfully raise a pup through weaning, dams must simultaneously balance the metabolic demands of traveling, foraging and producing an energy-rich milk (Schulz and Bowen, 2004) while minimizing consequences to pup health and growth.

**Table 1:** Published maternal attendance data are summarized (perinatal period in days, dam foraging trip duration in hours, and dam shore visit duration in hours) for the Steller sea lion breeding range, ordered northwest to northeast Pacific Ocean

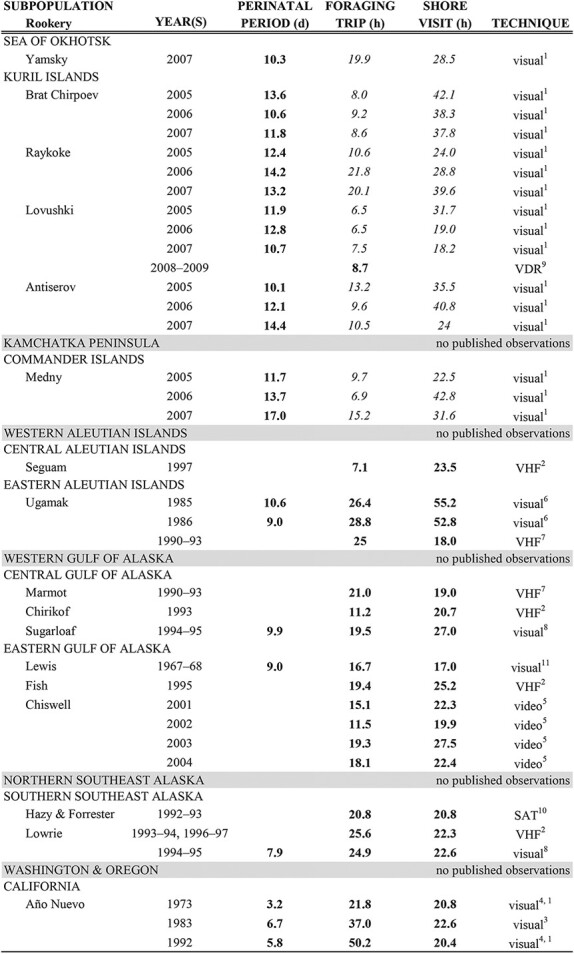

Mean values are presented in bold text, while median values are distinguished by italics. Data were obtained through five study techniques: visual observations at rookery (visual), through instrumentation of adult female SSL (VHF, SAT, VDR), or through remote video surveillance (video). Subpopulations shaded in light grey have no published quantified observations of maternal attendance metrics. Studies referenced in table: ^1^Burkanov *et al.*, 2011; ^2^Brandon, 2000; ^3^Higgins *et al.*, 1988; ^4^Hood and Ono, 1997; ^5^Maniscalco *et al.*, 2006; ^6^Merrick *et al.*, 1988; ^7^Merrick and Loughlin, 1997; ^8^Milette and Trites, 2003; ^9^Olivier *et al.*, 2022; ^10^Rehberg *et al.*, 2009; ^11^Sandegren, 1970.

Of these maternal attendance metrics, much effort has been dedicated to understanding variability in dam foraging trip duration (Table 1). Differences in dam age, pup age, rookery, year, local bathymetry and local prey availability have demonstrated significant influence on foraging trip duration (Higgins *et al.*, 1988; Hood and Ono, 1997; Maniscalco *et al.*, 2002; Milette and Trites, 2003; Maniscalco *et al.*, 2006; Burkanov *et al.*, 2011). Field studies designed to compute and compare maternal attendance metrics have contributed greatly to the understanding of a species’ life history and physiology. These studies are, however, logistically complicated (NRC, 2003), and in order to be comprehensive, often lengthy and cost-prohibitive. Methods have included relying upon on-site observers throughout the early breeding season (Sandegren, 1970; Higgins *et al.*, 1988; Hood and Ono, 1997; Brandon, 2000; Milette and Trites, 2003; Burkanov *et al.*, 2011), installation and maintenance of remote video surveillance systems (Maniscalco *et al.*, 2006) and deployment of VHF, satellite and/or video-depth recorder transmitters on adult lactating females (Merrick and Loughlin, 1997; Davis *et al.*, 2006; Rehberg *et al.*, 2009; Olivier *et al.*, 2022) to gather maternal attendance data.

### Biomarkers of metabolic status

**Figure 1:**
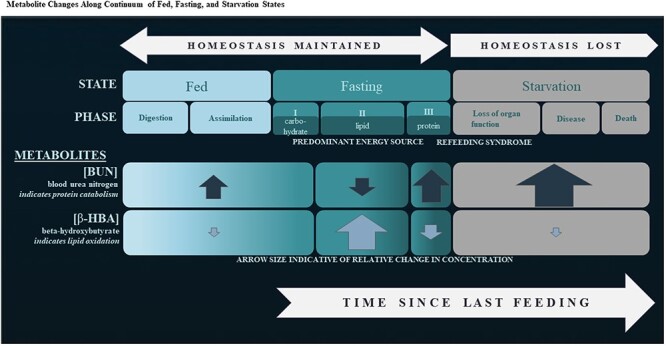
Illustration of an animal’s consumption states (fed, fasting, starvation) and the phases an animal progresses through during each state. With respect to the current study, changes in the plasma concentrations of blood urea nitrogen ([BUN]) and plasma concentrations of β-hydroxybutyrate ([β-HBA]) are indicated by arrow direction (up = increase, down = decrease in concentrations) and arrow magnitude. Homeostasis is maintained throughout both fed and fasting states; when homeostasis is unable to be maintained, a state of starvation ensues. Synthesized from Le Maho *et al.*, 1981; Cherel *et al.*, 1988; Costa, 1991; Castellini and Rea, 1992; Rea *et al.*, 2000; Verrier *et al.*, 2009; Champagne *et al.*, 2012; Lignot and LeMaho, 2012; Secor and Carey, 2016; Crocker and Champagne, 2018; Rosen and Worthy, 2018.

Blood of young pups on rookeries contain markers indicative of their fundamental metabolic state—feeding, fasting, or starvation (Figure 1). As post-prandial pups transition into a fasting state onshore during the dam’s foraging trips, they progress through three distinct fasting phases to maintain metabolic homeostasis by systematically catabolizing endogenous energy sources (Castellini and Rea, 1992; Secor and Carey, 2016), resulting in predictable changes in blood metabolite concentrations, as observed by Rea *et al.* (2000) in young SSL pups under experimental fasting conditions. Plasma-derived blood urea nitrogen concentrations ([BUN]) and plasma β-hydroxybutyrate concentrations ([β-HBA]) are two easily-measurable metabolites produced through the catabolism of proteins and lipids, respectively; the concentrations of BUN and β-HBA are expected to reflect the energy source predominantly utilized by the fasting pup (Secor and Carey, 2016).

### Distinctions among fasting phases

The primary macronutrient fueling pup metabolic demands is expected to change with fasting duration and characterizes the three phases of fasting (Figure 1). Following the departure of the dam to forage at-sea, pups digest and assimilate their most recent milk meal. In the absence of additional meals, *Phase I* begins, characterized by the catabolism of readily available carbohydrates (stored as glycogen in liver and muscle; Cherel *et al.*, 1988; Lignot and LeMaho, 2012; Secor and Carey, 2016). Pups who recently were fed or had begun *Phase I* fasting were expected to have elevated plasma [BUN], likely a result of the catabolism of both dietary and endogenous proteins (Rea *et al.*, 2000; Rosen, 2009; Secor and Carey, 2016) and very low plasma [β-HBA] (Robin *et al.*, 1988; Lignot and LeMaho, 2012).

Rea *et al.* (2000) subjected four 6-week old captive SSL pups to a 2½ day fast to assess the changes in metabolites over this period. Within 16 hours onset of this experimental fast, Rea *et al.* (2000) observed a transition of SSL pups into the protein-sparing phase, or *Phase II* of fasting, based on increased [β-HBA] concurrent to a decreased [BUN]. While ketone bodies can serve as a fuel source for much of the nervous system and some other cells, [BUN] did not decrease below detection limits, as gluconeogenesis likely continued, supporting certain portions of the central nervous system (Castellini and Rea, 1992). In the absence of refeeding, pup adiposity is the factor limiting the duration of *Phase II*. The percent total body lipids (%TBL) increases as pups age; an initial 3.4% mean TBL was observed in neonatal pups (1–5 days old; Davis *et al.*, 2006). A study of slightly older pups (1½–3 months old) showed increased %TBL, but also regional differences among the central and eastern Gulf of Alaska (15.1% TBL), Southeast Alaska (17.5% TBL) and central and eastern Aleutian Islands (21.8% TBL; Rea *et al.*, 2016).

When the captive fasting interval continued for 2½ days, plasma [β-HBA] and plasma [BUN] changed in SSL pups (Rea *et al.*, 2000), suggesting that pups reverted to catabolizing proteins to meet energetic demands prior to exhaustion of available lipid stores–thus entering *Phase III* fasting. During this transition from *Phase II–III,* [BUN] increased and the metabolic byproducts of lipid mobilization began to decrease (Rea *et al.*, 2000). Once pups fully transitioned into *Phase III*, [BUN] increased further and [β-HBA] decreased to levels observed prior to *Phase II* (Rea *et al.*, 2000). During *Phase II,* minimal protein is lost from lean body mass while predominantly lipids are catabolized (Verrier *et al.*, 2009). During *Phase III*, animals can transition from a fasting state into a starvation state that results in the rapid loss of lean body mass (Verrier *et al.*, 2012). While metabolite concentrations indicating *Phase III* status are rarely observed in free-ranging pinnipeds (Houser and Costa, 2003), an individual pup’s departure from *Phase II* and subsequent entry into *Phase III* is expected to have a strong relationship with pre-fasting levels of adiposity (Rea, 1995). Despite the favorable condition of SSL pups in the Rea *et al.* (2000) study (robust and in good body condition prior to the experimental fast; personal communication, Dr L. Rea, University of Alaska Fairbanks, 2020), metabolite concentrations changed over the 60-hour experimental fast suggesting that these pups had transitioned to *Phase III* during this relatively brief period.

There is an important distinction between the state of prolonged fasting and a state of starvation (Figure 1). During all phases of fasting, homeostasis is maintained and essential organ functions are supported (Castellini and Rea, 1992). Fasting, while expected, can only continue for a discrete period, the magnitude of which varies greatly due to the species’ adaptive capacity to fasting and an individual’s pre-fasting condition (Lignot and LeMaho, 2012). When endogenous energy stores (i.e. muscle and liver glycogen; subcutaneous, visceral and tissue fat; and amino acids from muscle) can no longer maintain homeostasis, starvation commences, potentially resulting in multiple concurrent and cascading organ failures that can progress to irreversible injury and ultimately death. While the state of starvation is unlikely to be reversed in free-ranging pups, *Phase III* can potentially be reversed following refeeding (Lignot and LeMaho, 2012); after significant time in *Phase III,* however, even refed pups may have a poor prognosis, eventually leading to mortality via starvation (Lignot and LeMaho, 2012; Rosen and Worthy, 2018). This failure to thrive once fed following a prolonged absence of food is well-documented in human (see reviews in Boateng *et al.*, 2010 and McKnight *et al.*, 2019) and domesticated animal medicine (Chan, 2015; Luethy *et al.*, 2020), as well as in some wildlife (Winn, 2006; Gerlach *et al.*, 2013; Fravel *et al.*, 2016; McRuer and Ingraham, 2019). Indications of refeeding syndrome include a combination of electrolyte imbalances, often characterized by hypophosphatemia, hypomagnesemia and hypokalemia (Crook *et al.*, 2001; Mehanna *et al.*, 2008; Boateng *et al.*, 2010; Crook, 2014). Possible associations with thiamine and other vitamin deficiencies (Crook *et al.*, 2001), as well as trace element deficiencies (e.g. selenium and copper, Boateng *et al.*, 2010) have been suggested as additive and causative agents. Cardiac and renal function are often compromised following a disruption of water balance and associated abnormalities in blood pressure (Crook *et al.*, 2001; Crook, 2014).

### Study importance

This study sought to investigate the variability of the fasting status of SSL pups born in different subpopulations, especially within discrete portions of their western range in the U.S., where the population abundance continues to decline (Holmes *et al.*, 2007; Fritz *et al.*, 2014; Altukhov *et al.*, 2015). During the last quarter of the 20th century, the detection of a precipitous decline in SSL population abundance prompted listing the species under the U.S. Endangered Species Act in 1990 (U.S. legal protections well-summarized in NRC, 2003). In 1997, two Distinct Population Segments (DPS) were delineated within the U.S. SSL range: the 1) western (wDPS; listed as endangered) and 2) eastern (eDPS; listed as threatened) (NMFS, 2013). Subsequent research and surveys determined that the eDPS SSL population had been steadily increasing for the past 40+ years at ~ 3% annually and the threatened status of the eDPS was removed in 2013 (NMFS, 2013). The decline observed throughout most of the wDPS ceased over the 1990s, and most subpopulations within the wDPS have been stable to modestly increasing in abundance for approximately the past two to three decades; there are some locations in the westernmost portion of the wDPS range, however, where the population continues to decline.

This study categorized individual pups into a discrete fasting category using concentrations of plasma BUN and plasma β–HBA, applying thresholds developed from Rea *et al.* (2000). We expected to identify pups along the entire fasting continuum illustrated in Figure 1. Due to variations reported in population trends throughout the SSL breeding range, we tested for differences in pup fasting duration at a finer geographic scale, groupings of rookeries commonly used for comparison by management authorities, hereafter referred to as *subpopulations*. We hypothesized that higher proportions of SSL pups with longer fasting durations would be present in subpopulations where population abundance has failed to recover.

## Methods

### Study area

**Figure 2:**
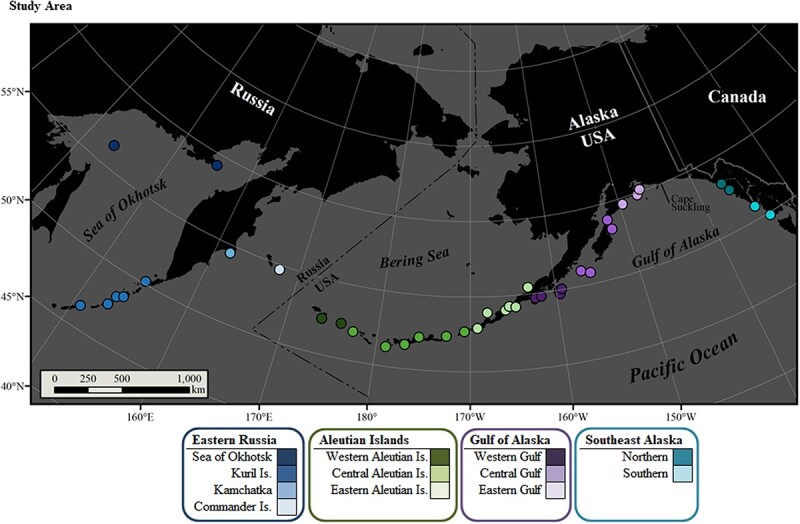
Steller sea lion natal rookeries (dots) sampled in this study. Pups from four regions were included: eastern Russia (blues), Aleutian Islands (greens), Gulf of Alaska (purples), and Southeast Alaska (aquas). The 12 subpopulations represented are color coded in different shades of the regional color. Within the USA, the designation at Cape Suckling separates the western and eastern Distinct Population Segments at 144° longitude.

This study utilized plasma samples collected during routine capture operations of SSL pups, born at 38 natal rookeries within four broad regions including eastern Russia and the Aleutian Islands, Gulf of Alaska and Southeast Alaska within the United States (Figure 2, Table 2, n = 1528, captured 1990–2016). Within these four regions, 12 subpopulations were represented in this study, moving eastward across the northern Pacific Ocean: within eastern Russia, the 1) Sea of Okhotsk, 2) Kuril Islands, 3) Kamchatka Peninsula and 4) Commander Islands and within the United States, the 5) western, 6) central, and 7) eastern Aleutian Islands, the 8) western, 9) central, and 10) eastern Gulf of Alaska, and the 11) northern and 12) southern portions of Southeast Alaska (Figure 2).

**Table 2:** Annual numbers (n) of plasma samples from individual Steller sea lion pups. Sampling breaks in the sequence of years are indicated by a thick grey line

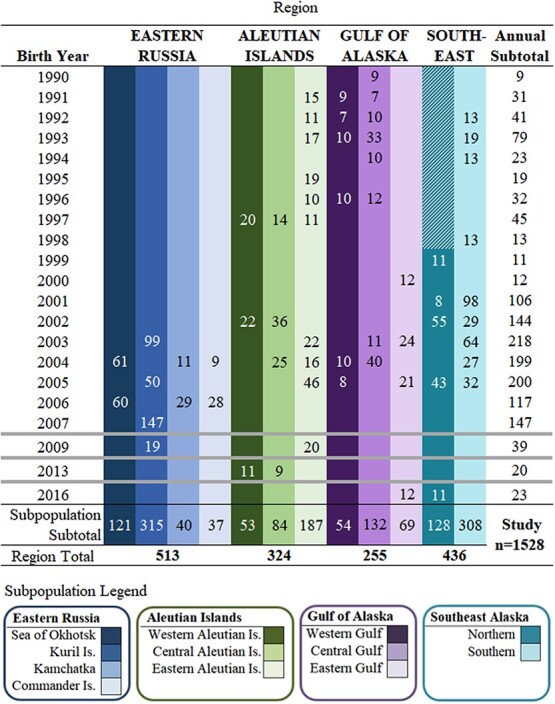

The striped area in the northern Southeast Alaska subpopulation represents years (1990–1998) that no samples could exist, as rookeries had not yet been established to create this subpopulation.

### Sample collection

Free-ranging SSL pups were physically (Brown *et al.*, 1995; Castellini *et al.*, 2012; Keogh *et al.*, 2013) or chemically restrained via gas anesthesia (Heath *et al.*, 1997; Haulena, 2014) at their natal rookeries (see Supplemental data for comparison of handling techniques). Captures within the United States were conducted by the Alaska Department of Fish and Game (ADF&G), National Atmospheric and Oceanic Administration (NOAA) National Marine Mammal Laboratory (NMML), and the Alaska SeaLife Center (ASLC) under authority granted through permits issued by the NOAA National Marine Fisheries Service (Marine Mammal Protection Act Permit #s: 809, 965, 14 325, 14 326, 18 528-00, 15 837-00, 358-1564, 358-1769, 358-1888, 782-1447, 782-1532, 782-1889, 782-1523, 881-1668, 881-18 900-02) and following Institutional Animal Care and Use protocol standards (ADF&G # 03-002, 06-07, 09-28, 2010-1412; ASLC # 07-01; Marine Mammal Laboratory # A/NW2010-4, A/NW 2013-2, A/NW 2016-3). Rookeries in eastern Russia were sampled in 2003–2007 and in 2009 with approval of Russian permitting agencies (Burkanov *et al.*, 2011). Blood samples obtained from the vein of the caudal gluteal plexus were drawn into blood collection tubes containing ethylenediaminetetraacetic acid or sodium heparin anticoagulants. Blood samples were kept chilled in the field until centrifuging (3000–3500 rpm for ten minutes), typically within four hours of collection (Rea *et al.*, 1998; Keogh *et al.*, 2013). Efforts were made to minimize disturbance to SSL on the rookery. Typically, individual pups were directly handled for 10–20 minutes for measurements, sample collection and/or tagging. Total rookery disturbance intervals (researchers’ arrival to departure) were typically < 8 hours. The time of blood collection with respect to the onset of rookery disturbance was not available for evaluation for many pups in this study. Plasma aliquots were stored at −20°C during the remainder of the field research trips (3–10 days) and at −80°C thereafter (months to years, maximal interval of 8 years) following return to the laboratory. Plasma samples were loaned to the University of Alaska Fairbanks (UAF) by the permitted agency for use in these analyses. The UAF Office of Research Integrity indicated that an additional Institutional Animal Care and Use protocol was not necessary as this laboratory project utilized archived tissue samples and did not directly involve handling live animals. During field physical examinations, morphometrics (mass, standard length, axillary girth) and sex were recorded for most pups, as well as any observed external abnormalities. Sampling dates ranged between May 26–August 26. Due to the semi-synchronous nature and geographic variation of parturition in SSL, pups on a rookery could have been within six weeks of age of each another (Pitcher *et al.*, 2001; Maniscalco and Parker, 2018).

### Metabolite assays

Plasma-derived [BUN] and [β-HBA] were measured via spectrophotometer (SpectraMax 340PC384, Molecular Devices, San Jose, CA) using commercially-available endpoint assay kits: [BUN] via StanBio Kit #2050 and Sigma Aldrich Kit #66–20 and #MAK006; [β-HBA] via StanBio Kit #2440 and Sigma Aldrich Kits #310 and #MAK001 (StanBio, EKF Diagnostics USA, Boerne, TX; Sigma Aldrich, now Millipore Sigma, St. Louis, MO) (Castellini and Costa, 1990; Castellini *et al.*, 1993). Plasma samples with moderate to severe hemolysis were not included in this study. Technical replicates were ≤ 10% coefficient of variation; assays were repeated for samples exceeding this threshold. Calculations of metabolite concentrations were made using Softmax Pro (v. 4.8) software or the open-access interface MyAssays.com, applying a four-parameter logistic curve. Beginning in 2016, a handheld ketometer (Precision Xtra™, Abbott Laboratories, Abbott Park, IL, ketometer precision = 0.1 mmol/L [β-HBA]), was used to identify plasma samples with [β-HBA] above and below a 0.3 mmol/L threshold needed to categorize fasting phase (Crawford *et al.*, In Review). Samples measuring between 0.2–0.4 mmol/L via ketometer were further analysed via the biochemical assay to improve precision around that threshold (assay precision≈0.01 mmol/L [β-HBA]). Earlier investigations in SSL pup fasting did not find predictable variation in concentrations of non-esterified fatty acids (Rea *et al.*, 1998) or glucose over the fasting period (Rea *et al.*, 2000). No other biomarkers were measured in the scope of this study.

### Fasting phase categorization

Thresholds defined for each fasting state were based upon the changes observed in plasma [BUN] and plasma [β-HBA] during an experimental fast imposed upon four captive six-week old SSL pups (Rea *et al.*, 2000; Table 3). Using these thresholds, one of four fasting categories was assigned to each individual pup: 1) Recently Fed–Phase I fasting (*Fed–I*), 2) Phase II fasting (*II*), 3) transitioning between Phases II and III fasting (*II–III*), 4) Phase III fasting (*III*). Using additional information available for 87 SSL pups, *a priori* expectations of fasting classifications were compared to the classification assigned using metabolite thresholds. This additional information was obtained via field records identifying individuals as neonates, emaciated/starveling/orphaned, skinny/thin, or having milk in their stomachs (sampled via gastric lavage during another study, Beckmen *et al.*, 2016).

**Table 3:** Thresholds used to assign fasting phase of Steller sea lion pups, based on changes in concentrations of blood urea nitrogen (BUN) and β-hydroxybutyrate (β-HBA) measured in plasma during a fast imposed upon 6-week old captive Steller sea lions (Rea *et al.*, 2000)

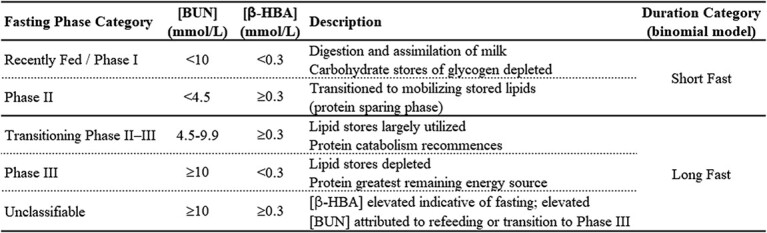

### Data selection

Quantitative analyses were conducted using open-source software R (R Core Team, 2019). Criteria for inclusion in this study required a minimum of five pups sampled on each location/date combination to assess the proportion of pups in different fasting phases (Table 2). Previously published results from 16 pups sampled during 1990 and 1991 in the Gulf of Alaska (Castellini *et al.*, 1993) and an additional 220 pups sampled in Southeast Alaska, the Gulf of Alaska and the Aleutian Islands between 1991–1996 (Rea *et al.*, 1998) were included in this study for comparison.

Maniscalco *et al.* (2006) identified an inflection point in the maternal attendance cycle when dam foraging trip durations significantly increased, occurring around July 19^th^ at the Chiswell rookery (eastern Gulf of Alaska). Before including pups sampled after July 19^th^ of any year into the current study, a Kruskal–Wallis nonparametric one-way ANOVA on ranks (stats R package) was used to compare the proportion of pups categorized as *Fed–I* phase before and after July 19th of any year for subpopulations where such late summer samples were collected.

### Body condition indices

Body condition indices have proven more useful for assessing Otariid condition than individual morphometrics, as SSL are a highly sexually dimorphic species, even distinguishable as young pups (Higgins *et al.*, 1988; Brown *et al.*, 1995; Ono and Boness, 1996). Though there are multiple published calculations of body condition applied to SSL pups, this study used an index detailed by Trites and Jonker (2000):\begin{align*}&\text{Body condition index}\\ &\quad=\left(\frac{body\ mass\ (kg)}{-63.88+0.8966\times dorsal\ standard\ l\mathrm{e} ngth\ (cm)}\right).\end{align*}

### Data analysis

Initial within fasting category comparisons were made at the subpopulation level using Kruskal–Wallis nonparametric tests, followed by a Dunn test for multiple comparisons and using a Bonferroni correction (*FSA* R package). To identify differences in pup fasting duration among subpopulations, we further classified pups into *Short* or *Long* fast categories. Pups initially characterized as *Fed–I* or *II* were combined into the *Short* category; all other pups were combined into the *Long* category (Table 3). This allowed us to fit a binomial regression to assess differences attributed to pups in *Short* or *Long* fasts with respect to body condition index, sex and/or subpopulation (Warton and Hui, 2011; *stats* R package). Using the categorical response variable of fasting duration (*Short* or *Long*), the global model included the fixed-effects of the body condition index, subpopulation, sex, timing of sampling (before or after July 19) and interactions between variables. The top model was selected using a backward selection process. Statistical significance was identified using α < 0.05. Parameter estimates were calculated for the marginal means (95% confidence interval). Tukey contrasts were used to assess differences between each combination of the 12 subpopulations. Post-hoc Chi-square tests were used to test for differences in sex distribution within fasting phases with respect to subpopulation (*stats* R package).

Statistical comparisons for interannual variability in fasting state across the breeding range, within subpopulation, or within rookery were not made, due to few rookeries with robust, multi-annual sampling regimes (Table 2). Unfortunately, the resulting dataset was discontinuous and unbalanced and not appropriate for statistical analysis as a time series.

## Results

Following review of the capture records for pups included in this study, we concluded that the vast majority of pups appeared to be clinically normal as determined by external physical examination. Exceptions are described below in our evaluation of *a priori* expectations. Our comparison of metabolite concentrations in chemically- and physically-restrained SSL pups identified no significant differences attributable to either anesthesia or manual restraint (see Supplemental data).

Our assignment of concentration thresholds of plasma metabolites among categories of fasting were in most cases validated, using a subset of pups with documented conditions (poor body condition, presence of milk in stomach at time of capture; Table 4). Pups noted as emaciated/starveling/orphaned (n = 10) were all classified as either *Phase II–III* or *III,* fully meeting our expectations, while three pups described as skinny/thin were categorized as *Fed–I*. Pups identified as neonates were expected to be classified as *Fed–I* (n = 33), but only 59% of cases matched expectations. Similarly, only 58% of the pups identified with stomachs containing milk were categorized as *Fed–I* (n = 27). Pups observed with dam via video surveillance within 8.2 hours prior to blood sampling met our expectations of *Fed–I* (Figure 4). However, our expectations–based upon the pup metabolite concentrations supporting an entrance into *Phase II* within 16 hours of fasting onset (as observed by Rea *et al.*, 2000)–were not met for the interval 8.2–16 hours. Pups with a last known observation with the dam ranging from 16–48 hours only met expectations in two of five cases (Table 4).

**Table 4:** Comparison of *a priori* expected fasting phase classifications to outcomes following application of the thresholds (Table 3) for a subset of pups with field records indicating a certain status (n = 87)

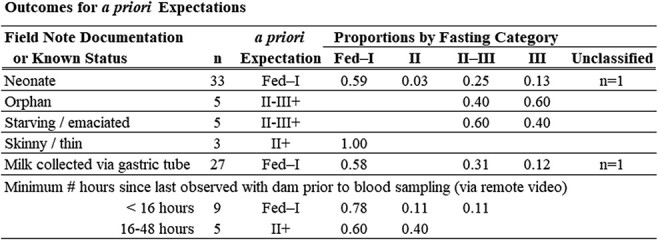

**Figure 3:**
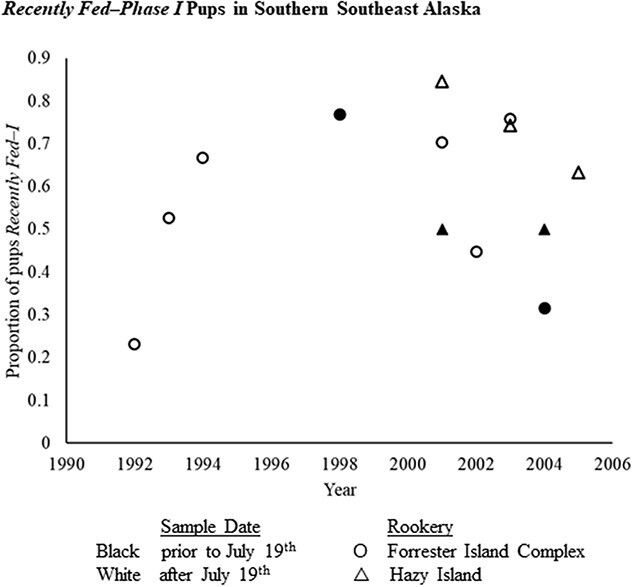
Proportion of Steller sea lion pups categorized as *Recently Fed–Phase I* fasting for plasma samples collected before (open symbols) and after (closed symbols) July 19^th^ at two rookeries in the southern Southeast Alaska subpopulation: Forrester Island complex (circles) and Hazy Island (triangles). In the Gulf of Alaska, July 19^th^ was found to be an inflection point at which adult females began extending the duration of their foraging trips (Maniscalco *et al.*, 2006).

Before applying the binomial model, fasting phase distributions in the three subpopulations with pups sampled post-July 19^th^ of any year were examined. No significant differences in the proportion of pups categorized as *Fed–I* were observed between pups sampled post-July 19^th^ in northern Southeast Alaska (*Fed–I* proportion = 0.70, n = 19) or in the eastern Gulf of Alaska (*Fed–I* proportion = 0.83, n = 12) when compared to all other pre-July 19^th^ samples (*Fed–I* proportion = 0.72, n = 1468). The southern Southeast Alaska subpopulation, however, exhibited a significantly lower proportion of pups in *Fed–I* in this same comparison (*Fed–I* proportion = 0.38, n = 29; Kruskal–Wallis: H = 10.72, *P* < 0.01). Further comparisons between pups from two southern Southeast Alaska rookeries, Forrester Island Complex and Hazy Island, sampled between June–August indicated that the post-July 19^th^ proportions of *Fed–I* were not significantly different than those observed pre-July 19^th^ within this subpopulation (Kruskal–Wallis: H = 1.62, *P* = 0.445, Figure 3). Finding no evidence of longer dam foraging in these areas, data for all young pups were included in subsequent analyses.

Of 1528 pups included in this study, only 21 (1.4%) were unable to be categorized into one of the four discrete fasting categories. Each unclassifiable pup had elevated [β-HBA] (indicative of lipid oxidation) and [BUN] (indicative of protein catabolism); we therefore categorized these 21 pups into the *Long* fast category for inclusion in the binomial model only. We observed a mean plasma [BUN] of 5.7 ± 0.07 mmol/L and a mean plasma [β-HBA] of 0.23 ± 0.004 mmol/L for all SSL pups in this study combined (Figure 5).

### Within-fasting category comparisons

The majority of pups in each subpopulation were classified as *Fed–I* (Figure 6A); a grand mean proportion of 0.72 within *Fed–I* was observed (n = 1077). The greatest proportion of pups in *Fed–I* was observed in both the Kamchatka Peninsula and western Gulf of Alaska subpopulations (proportion = 0.83), while the lowest proportion of pups observed in *Fed–I* was in the western Aleutian Islands (proportion = 0.57). A significant difference among subpopulations was detected within the *Fed–I* category (Kruskal–Wallis χ^2^ = 29.502, df = 11, *P* = 0.0019); post-hoc tests identified no specific significant differences. No significant differences were identified among subpopulations within *Phase II* of fasting (Kruskal–Wallis χ^2^ = 16.935, df = 11, *P* = 0.1098); the range of proportions was 0.02–0.12 among subpopulations, representing 133 individual SSL pups (Figure 6B). Notably, all subpopulations had higher proportions of pups categorized as transitioning *II–III* (n = 242 individual SSL pups) than *Phase II*. Significant differences were detected for pups transitioning between *II-III* (Kruskal–Wallis χ^2^ = 44.903, df = 11, *P* < 0.0001). While three subpopulations had more than 20% of pups in the transition phase (the central and western Aleutian Islands and southern Southeast Alaska), post-hoc tests revealed that the western Aleutian Islands (Dunn test with Bonferroni adjustments Z = -3.8538, *P* = 0.0077) and southern Southeast Alaska (Dunn test with Bonferroni adjustments Z = -5.2350, *P* < 0.0001) pups were significantly more prevalent in this transition phase than the eastern Aleutian Islands (Figure 6C). Southern Southeast Alaska also had a higher proportion of pups in *II–III,* when compared to the Kuril Islands (Dunn test with Bonferroni adjustments Z = -3.7774, *P* = 0.0105). No significant differences were identified among subpopulations within *Phase III* of fasting (Kruskal–Wallis χ^2^ = 14.800, df = 11, *P* = 0.1919; Figure 6D). *Phase III* had the lowest proportions of pups observed (4% of total observations, n = 55), and two subpopulations in Russia were unrepresented within *Phase III* (Kamchatka Peninsula and Commander Islands).

**Figure 4:**
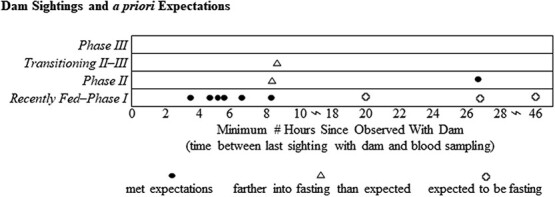
Fasting phase classification of Steller sea lion pups by number of hours since observed with adult female via video camera surveillance at Chiswell Island rookery, eastern Gulf of Alaska in 2016. Black dots indicate classifications for individual pups that met *a priori* predictions (Table 4). Triangles indicate pups classified farther into the fasting process, and those not fasting when a fasting state was expected are indicated by open circles.

**Figure 5:**
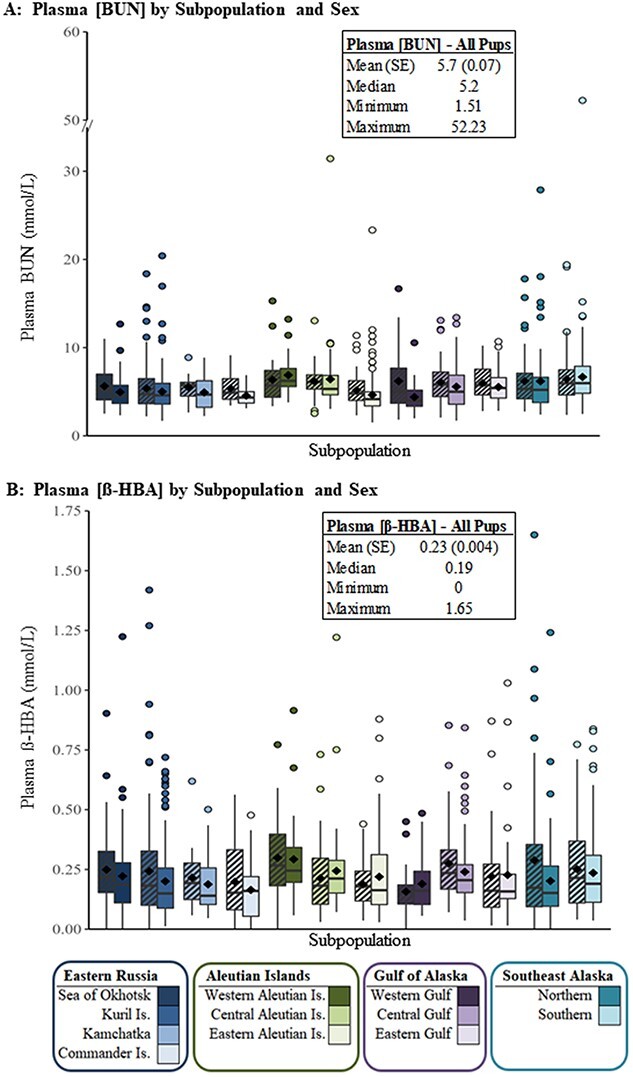
(A) Plasma [BUN] and (B) plasma [β-HBA] for 1528 SSL pups by 12 subpopulations and sex (females striped, male solid bars). The black diamond within each boxplot represents the mean observed concentration for that subpopulation and sex.

**Figure 6:**
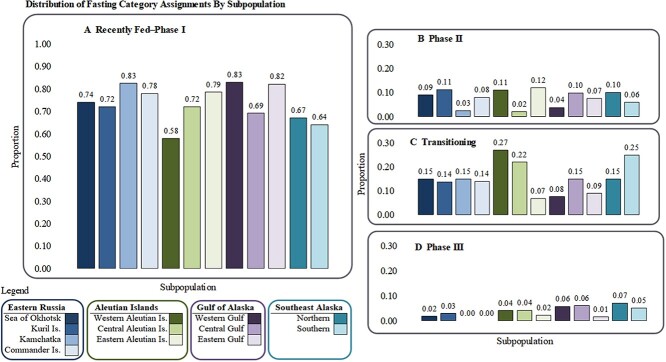
Proportion of pups categorized as (A) Recently Fed/Phase I fasting, (B) Phase II, (C) Transitioning Phase II–III, and (D) Phase III for each subpopulation, organized from the northwestern to northeastern Pacific Ocean. Subpopulations significantly different from others within each fasting category are denoted by lowercase letters. Note, the y-axis in (A, range 0.0–1.0) is different than that shown for (B), (C), and (D) with range 0.0–0.3. Sum of proportions for each subpopulation in (A), (B), (C), and (D) sums to 1.

### Binomial model of fasting duration

**Figure 7:**
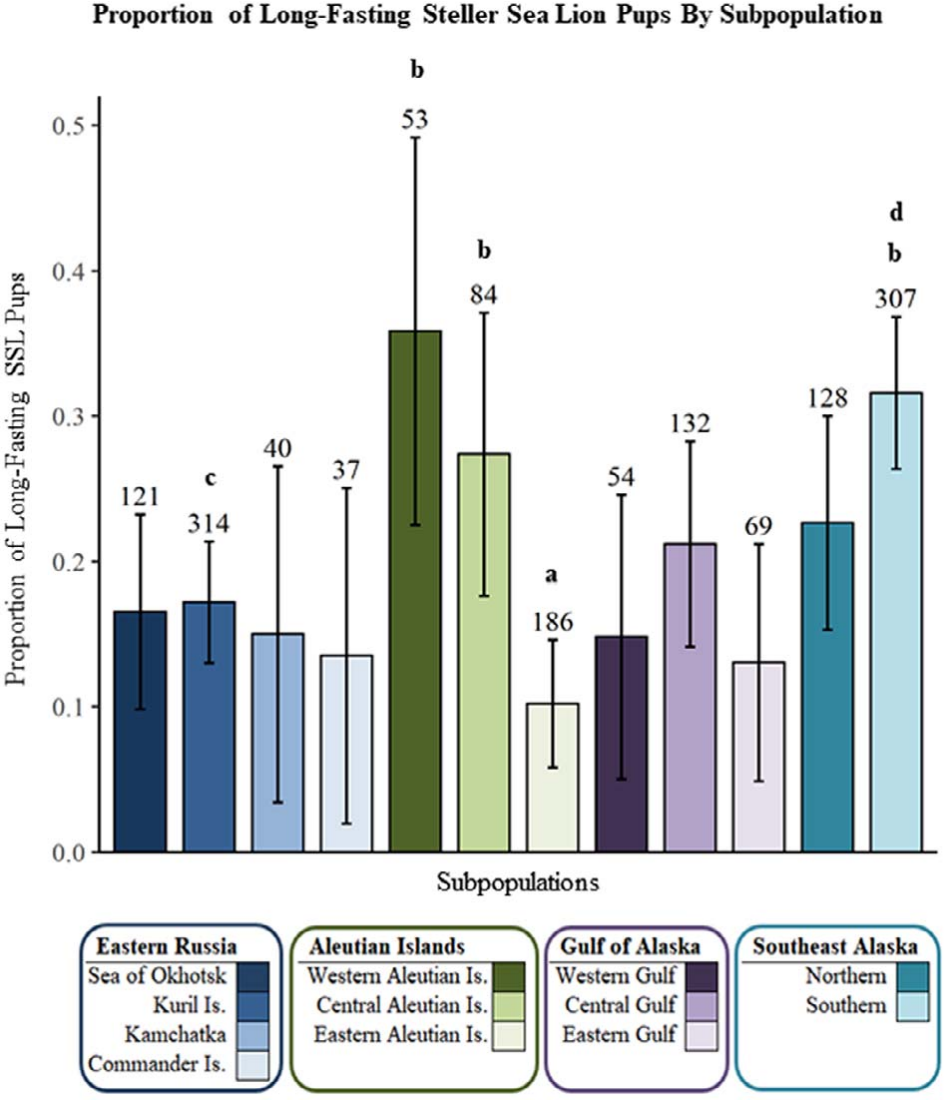
*Long Fast* duration binomial model parameter estimates by subpopulation, corrected for sex and ordered from northwestern to northeastern Pacific Ocean. Sample sizes are displayed above error bars. Subpopulations labeled “a” were significantly different than those labeled “b”, and those labeled “c” were significantly different from those labeled “d” (*P* < 0.03).

The most parsimonious model identified both sex (Z = 2.8987, *P* = 0.0038, df = 1524) and subpopulation (Z = 3.0409, *P* = 0.0024, df = 1524) as highly significant explanatory variables for fasting duration (Top model, Table 5). The parameter estimates for the proportion of *Long* fasts with 95% confidence intervals for each subpopulation, corrected for sex are reported in Table 6. The parameter estimates within the three Aleutian Island subpopulations included both the highest (western) and lowest (eastern) proportions of *Long* fasting pups, within a relatively small portion of the overall SSL breeding range. Only four of 66 contrasts were highly significant (Tukey, *P* < 0.03 for all four; Figure 7): the eastern Aleutian Island subpopulation had a lower proportion of *Long* fasting pups than the 1) western Aleutian Islands, 2) central Aleutian Islands and 3) southern Southeast Alaska subpopulations; the Kuril Islands also had a significantly lower proportion of *Long* fasting pups in contrast to the 4) southern Southeast Alaska subpopulation.

**Figure 8:**
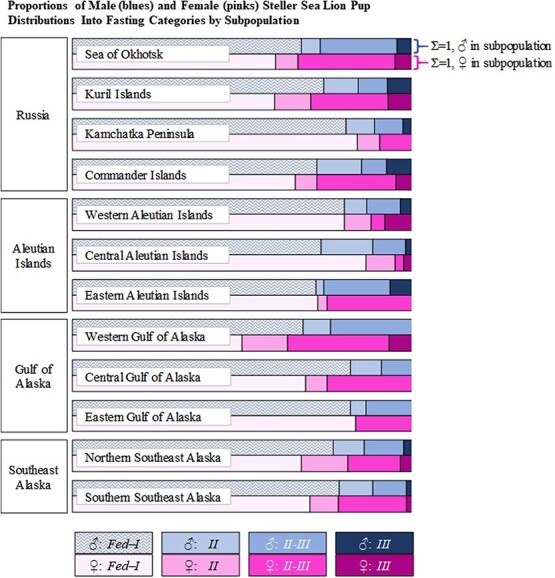
Comparison of the proportions of male (blues) and female (pinks) SSL pups distributed into the four fasting categories by subpopulation (white labels over each pair of rows). The sum of the proportions for each row equals one, standardizing comparisons of the frequency of pups within each fasting category between sexes.

While a significantly greater proportion of female pups were categorized into a *Long* fasting durations, no post-hoc comparisons were significant when comparing distribution into fasting phases by subpopulation with respect to the observed sex ratios (Table 7). The overall observed sex ratio was 54♂:46♀. Observed sex ratios within each subpopulation followed the same skew towards more males in all but two cases: a 50:50 sex ratio was observed in the Sea of Okhotsk, and the western Aleutian Islands had more female pups than males (45♂:55♀, Table 7). Though no statistical differences between sexes for *Long* fasting pups by subpopulation were identified, Figure 8 illustrates the magnitude of the variability between the proportions of all males/subpopulation and the proportions of all females/subpopulation categorized into the four fasting categories.

## Discussion

### Key findings

As expected, the majority of pups in this study and within each subpopulation were categorized as *Fed–I* and having *Short* fasting durations, as the means and medians of the [BUN] and [B-HBA] were relatively low (Figure 5). Consistent with other pinniped fasting studies, very few pups were classified into *Phase III* (Houser and Costa, 2003), as anticipated for a fasting-adapted species that tends to extend *Phase II* (Verrier *et al.*, 2009). Also, as expected, pups were distributed into all categories described across the fasting continuum (Figure 1).

Both the within-fasting category comparisons and the binomial model identified the same three subpopulations with greater proportions of pups in the more prolonged fasting categories: 1) the western and 2) central Aleutian Islands and 3) southern Southeast Alaska subpopulations. These areas of the Aleutian Islands and Southeast Alaska experienced divergent population growth patterns for the past 50 years: SSL populations in Southeast Alaska (eDPS) steadily increased (Allen and Angliss, 2013), while populations in the western Aleutians and portions of the central Aleutians continued to decline (Holmes *et al.*, 2007), suggesting support for both density-dependent and -independent influences on the duration of fasting periods.

Findings from the increasing population in the eDPS support the potential for density-dependent influences in this area. SSL pups from the eDPS had reduced body masses (Brown *et al.*, 1995; Rea *et al.*, 1998; Brandon *et al.*, 2005; Hastings *et al.*, 2020*a*), and young-of-the-year eDPS SSL had reduced body mass and percent total body lipids (Rea *et al.*, 2016), as compared to the wDPS. The mean dam foraging trip durations in the eDPS were amongst the longest observed (Brandon, 2000; Milette and Trites, 2003). Pups from eDPS weaned later than those from the wDPS, with exception of pups from the eastern Gulf of Alaska subpopulation (Hastings *et al.*, 2020*a*). Three new rookeries were established (northern Southeast Alaska subpopulation in our study; Mathews *et al.*, 2011), and more recent genetic investigations have elucidated more complex dispersive movements within the eDPS and between the eDPS and wDPS than was previously understood (Jemison *et al.*, 2013, 2018; Hastings *et al.*, 2020*b*).

### Addressing potentially confounding factors

There are few studies on fasting blood chemistry of free-ranging Otariid pups upon which to compare our approach and findings (Castellini *et al.*, 1993; Rea *et al.*, 1998; Arnould *et al.*, 2001; Verrier *et al.*, 2011, 2012). The majority of the research measuring metabolite concentrations on young pinniped pups has been conducted on Phocids, which exhibit considerable differences in life history as neonates through weaning, complicating comparisons. Phocid pups have a single intensive nursing period prior to weaning (varying between 4 days to 7 weeks among Phocid species); there is not an alternation between fasting and fed states as in Otariid pups (Crocker and McDonald, 2016). Furthermore, we established our thresholds used to assign fasting category in this study, based upon results from the Rea *et al.* (2000) 2½ day experimental fast imposed on four captive 6-week old SSL pups. Plasma samples collected from free-ranging SSL pups are limited to providing a snapshot view of pup metabolite status. Our interpretation of these data is limited as we lack additional information about pups’ prior fasting histories, pre-fasting adiposity, or specific birthdate.

**Table 5:** Variables evaluated using backward selection for the binomial model comparing *Short* and *Long* fasts

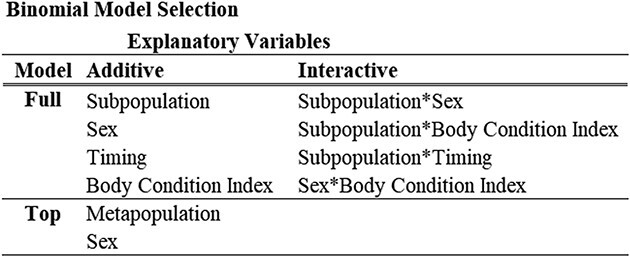

**Table 6:** Parameter estimates and 95% confidence interval for the mean proportion of pups categorized as *Long* fasting duration by subpopulation from the binomial regression model

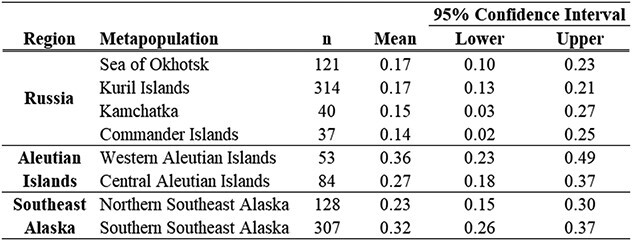

**Table 7:** Proportion of ♂ pups by subpopulation and overall, within each fasting of four fasting categories, and classified into *Long* fasting duration

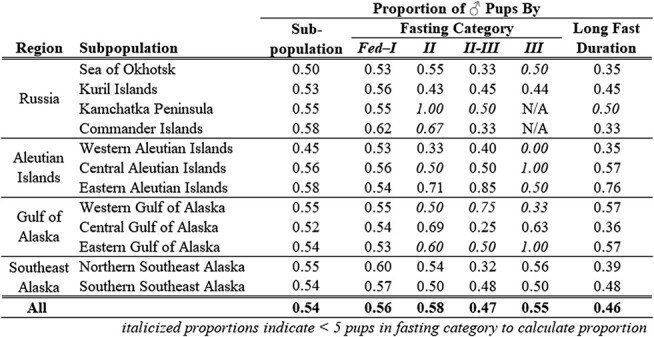

*Italicized values* indicate that the proportion was calculated from < 5 individual SSL pups assigned into that category for that subpopulation.

Variations in metabolic analytes may result from physiological responses to other scenarios than fasting. In a clinical setting, a differential diagnosis for SSL pups with elevated [BUN] should minimally include diagnostics for dehydration, infection of *Leptospira* spp., reduced cardiac function, renal function and glomeruli filtration rate, amyloidosis and bleeding within the gastrointestinal tract (Chinnadurai *et al.*, 2008; Colegrove *et al.*, 2009; Field *et al.*, 2018; Lawler *et al.*, 2021). In the absence of fasting, elevated [β-HBA] would likely be associated with chronic pancreatitis or other conditions impacting the function of the pancreas (Colegrove, 2018; Field *et al.*, 2018). A comparison of serum chemistry panels of clinically-normal and -abnormal California sea lion (CSL; *Zalophus californianus*) of all age classes (undergoing rehabilitation at the Marine Mammal Center, Sausalito, California), found serum [BUN] to be significantly lower for CSL diagnosed with parasite infestation or verminous pneumonia and higher in CSL diagnosed with both pneumonia and gastrointestinal infections or septicemia related to peritonitis due to perforated gastric/duodenal ulcers, as compared to the clinically-normal animals (Roletto, 1993); results were not subset with respect to age class. In a study of serum chemistries of both wild and stranded CSL with known morbidities, a marked increase (6.5-fold) in [BUN] in animals with *Leptospira* infection was identified, but no difference in [BUN] was noted in those diagnosed with malnutrition, pneumonia, or trauma (Williams, 2013). Studies, such as those referenced above, involved wild pinnipeds evaluated in rehabilitation centers, available for additional sample collections and other diagnostic tests to evaluate their condition and further validate the diagnosis.

Significant exposure to *Leptospira* by SSL was not supported by serologic investigations (Burek *et al.*, 2003; 2005). Further, prior studies of SSL pup blood chemistry, hematology and metabolites all support that free-ranging pups maintain adequate hydration (Castellini *et al.*, 1993; Rea *et al.*, 1998; Lander *et al.*, 2013). Fasting-adapted pinnipeds maintain water balance during fasting through water contributions from lipid catabolism (Crocker and Champagne, 2018). While there is some overlap between pups evaluated in the aforementioned SSL studies and the current study, these additional data were not available to be evaluated for all pups with metabolite measurements. The field physical examinations of SSL pups included in this study indicate that nearly all were externally physically normal, with exception of those noted as skinny, thin, emaciated, starving, or orphaned (n = 13); these individuals were used in our evaluation of fasting phase categories (Table 3). Further, our analysis of a subset of SSL pups included in this study found no differences between pups that were physically and chemically restrained prior to blood sampling (see Supplemental data).

Some plasma samples in this study were archived at −80°C for several years (maximal interval of 8 years) prior to analyses; no available studies assess plasma metabolite stability at this storage temperature and duration. Any degradation of plasma samples that may have occurred during storage would likely result in reduced concentrations of the two metabolites we evaluated; this would conservatively result in a bias towards classification into *Short* rather than *Long* fasts. The serum [BUN] was found to be stable following storage at −80°C for 28 weeks after collection from CSL (Williams, 2013). Our internal evaluation identified no anticoagulant or freeze–thaw effects on SSL plasma [β-HBA] (Crawford *et al*., In Press). Other studies evaluating stability of rat or bovine plasma [BUN] or [β-HBA] with respect to anticoagulant (EDTA, heparin), storage time (up to one month), temperature (minimal −40°C) and freeze–thaw events also found no significant changes in metabolite concentration (Stokel and Nydam, 2005; Singh *et al.*, 2014). Additionally, a nuclear magnetic resonance study of bovine plasma samples stored for up to 15 years at −20°C found no significant difference in [BUN] or [β-HBA] (Trabi *et al.*, 2013).

### Evaluating the thresholds used in fasting categorization

With respect to our *a priori* expectations of fasting category (Table 4) for the subset of pups (n = 87) with additional information to inform our hypotheses, we have considered many explanations for the cases that met or failed to meet our expectations. The words used by field biologists and veterinarians to describe more dire pup conditions (i.e. emaciated, starveling, orphaned) were associated with pups that met our *a priori* expectations, reaffirming our interpretation of the field notes. Less specific descriptors (i.e. skinny, thin) were likely applied more subjectively in the field, and our interpretation and expectation of fasting category did not meet the outcomes. We were surprised to find that only 59% of pups identified as neonates were categorized as *Fed–I*; these pups were expected to be with the dam and still within the perinatal period. Further, we anticipated that the field identification of neonates would be highly accurate, based upon presence and condition of a retained umbilicus. The well-documented variability in the duration of SSL perinatal periods (Table 1), as well as the frequency/volume of feedings during the perinatal period (largely unknown or undocumented) could explain why our expectations were not fully met.

The evaluation of pups with milk in their stomachs at the time of handling illustrates the need for a more detailed understanding of the turnover time for the metabolites we evaluated, especially through the post-prandial into post-absorptive states. Similarly, the video surveillance record indicated that dams and pups were in each other’s proximity, but not specifically that nursing occurred at that time. The dam’s exact time of departure from the rookery was not available for consideration. Additionally dam-pup reunions could have occurred outside of the video frame.

While our expectations were not fully met in comparison to our *a priori* expectations, we found reasonable explanations for the discrepancies and no consistent differences in classification that would indicate that the thresholds utilized to assign fasting category need revision.

### Potential drivers for extended foraging trip durations

Our study approach was not designed to identify *causes* of variability in fasting duration, but rather to examine the existence of variability across much of the SSL breeding range. Together with management authorities, we can now investigate the variability detected in pup fasting duration in some subpopulations more thoroughly. As Otariid dams function as central place foragers during lactation, we suggest the simplest proximate explanation for longer pup fasting intervals is an increased duration of dam foraging trips, which further suggests an overall increased foraging effort (foraging effort includes all aspects of foraging–traveling, searching, pursuing, catching, consuming). The ultimate factors, however, that influence foraging duration likely include one or more variables related to local conditions (i.e. prey availability, competition, predation) and/or the health of an individual SSL. Studies of many of these factors exist in the literature as researchers extensively searched for the cause(s) of the wDPS SSL population decline that began in the 1970s (NMFS, 2008). We submit that each of these aspects affecting SSL life history are worthy of consideration; non-lethal and/or sub-clinical interactions may have significant effects on dam foraging behavior, which may manifest as measurable effects to pup health, body condition and viability.

The available prey base composition may vary in species abundance, diversity, distribution and/or quality. Previous pinniped maternal attendance studies have found that dams extended their foraging trips when prey resources were limited or had an atypical geographic distribution (i.e. during El Niño southern oscillations); this phenomenon is well summarized for Otariids by Milette and Trites (2003), Trites and Donnelly (2003), Maniscalco *et al.* (2006) and Burkanov *et al.* (2011). Understanding local trophic structure is complicated by the cascading effects of dynamic oceanographic regimes and climate change which can influence timing of phytoplankton blooms, fish distribution, winter ice extent in adjacent waters and storm frequency and intensity (Benson and Trites, 2002; Peña *et al.*, 2019; Hipfner *et al.*, 2020; Gulland *et al.*, 2022). The nutritional stress hypothesis was suggested as an explanation for the continued population decline in some portions of the SSL range (Calkins and Goodwin, 1988). While studies have produced mixed results, empirical evidence does not support that nutritional stress was a leading cause (see Fritz and Hinckley, 2005; Atkinson *et al.*, 2008; and Trites, 2021 for discussion of nutritional stress hypothesis studies).

Several sympatric piscivores, including other pinnipeds, cetaceans, sharks and seabirds, have dietary overlap with SSL. Conspecific competition may be significant in some portions of the SSL’s range. The impact of commercial fisheries has also been debated as a source of competition to SSL (see NRC, 2003 and Atkinson *et al.*, 2008 for a thorough review of fisheries and related studies on SSL). While some suggest that commercial fisheries were not a significant factor in the initial population decline of SSL (Hennen, 2006), others suggest that changes in fish biomass and/or fish size class structure due to commercial fishing have effectively altered local carrying capacity for some meso/apex predators (McHuron *et al.*, 2020).

The presence of predators has a well-known effect of increasing vigilance behaviors which consequently subtract time allocated towards other behaviors, such as foraging (Ripple *et al.*, 2001, 2016; Fortin *et al.*, 2005; Wirsing *et al.*, 2008; Ripple and Beschta, 2012; Suraci *et al.*, 2016; Gaynor *et al.*, 2019; Palmer *et al.*, 2022). Killer whales (*Orcinus orca*) are known predators of SSL (Barrett-Lennard *et al.*, 1995; Saulitis *et al.*, 2000; Heise *et al.*, 2003; Maniscalco *et al.*, 2007; Permyakov and Burkanov, 2009; Dahlheim and White, 2010). Prior energetics studies even modeled the potential for killer whales to be solely responsible for the decline of the wDPS SSL, as part of the hypothesis regarding a large megafauna collapse in the north Pacific Ocean in the last century (Springer *et al.*, 2003, 2008; DeMaster *et al.*, 2006; Estes *et al.*, 2009, 2011, 2016). More recent studies have identified a functional range expansion of killer whales associated with absence of sea ice (Higdon and Ferguson, 2009; Lefort *et al.*, 2020); increasing availability of suitable habitat and prey resources could result in increased killer whale populations and/or changes in their seasonal distribution adjacent to sea lion rookeries. Fate studies of juvenile SSL and other studies have provided convincing evidence of predation on SSL by Pacific sleeper sharks (*Somniosus pacificus*; Sigler *et al.*, 2006; Horning and Mellish, 2014; Bishop *et al.*, 2019). It is plausible that predatory pressure may have been a significant contributory factor in the decline or lack of recovery of SSL in some subpopulations, both directly and indirectly through increased vigilance and avoidance behaviors, culminating in measurable changes in dam foraging behavior (Wirsing *et al.*, 2008; Palmer *et al.*, 2022).

The impacts of morbidities caused by environmental contaminants are very difficult to demonstrate, quantify and interpret. Organochlorines, heavy metals and plastics have been identified in marine wildlife within the SSL’s range; collectively these contaminants are categorized as neurotoxins, carcinogens and/or endocrine disruptors. Several studies have identified an elevated presence of organochlorines in SSL tissues (Myers *et al.*, 2008; Wang *et al.*, 2011; Beckmen *et al.*, 2016; Keogh *et al.*, 2020). Additionally, higher proportions of elevated total mercury concentrations have been found in SSL pups (Rea *et al.*, 2020) and some fish (Cyr *et al.*, 2019) within the same discrete portion of the Aleutian Islands (western and central Aleutian Islands) where we identified greater proportions of pups in *Long* duration fasts. A sublethal impact to the acute phase inflammatory response of the immune system was found to be depressed in free-ranging SSL pups with higher total mercury concentrations (Kennedy *et al.*, 2019). Plastics have been observed in sediments, invertebrates and vertebrate species in the subarctic North Pacific (Doyle *et al.*, 2011; Desforges *et al.*, 2015; Mu *et al.*, 2019*a*, 2019b; Padula *et al.*, 2020; Zhang *et al.*, 2020). Though detailed, controlled studies on morbidities related to varying exposures of these contaminants to SSL or other pinnipeds have not been conducted, sublethal effects of contaminants on behaviors such as foraging should be considered as potential factors influencing dam foraging trip duration. For example, reduced foraging efficiency associated with increased dietary or maternal-derived mercury exposures have been documented across vertebrate species, including fish (Fjeld *et al.*, 1998; Grippo and Heath, 2003; Armstrong *et al.*, 2020), reptiles (Chin *et al.*, 2013) and birds (Adams and Frederick, 2009; Bouton *et al.*, 2009; Swaddle *et al.*, 2017; Grunst *et al.*, 2023).

### Breeding female age structure and pup adiposity

Prior studies have shown that maternal attendance factors were influenced by the dam’s parity history and age (Maniscalco *et al.*, 2002, 2006; Milette and Trites, 2003; Burkanov *et al.*, 2011; Hastings and Jemison, 2016). Burkanov *et al.* (2011) found that younger dams had perinatal periods of approximately 30% shorter duration than dams > 6 years of age on rookeries in Russia. These studies suggest that dam age, prior experience and condition would be positively correlated with pup condition and would therefore influence pup fasting physiology. Population assessments have considered adults, juveniles and pups separately, but little information is available about the age-structure of breeding females at the subpopulation level.

Pups with greatly reduced adiposity could move in an accelerated fashion through Phases *I* and *II* into the later fasting categories (*II-III, III*), resulting in a metabolite profile of a *Long* fasting pup over a shorter elapsed time than expected. Such a scenario could develop following subsequent long fasts, but without prior knowledge of a pup’s fasting history and condition, we are unable to evaluate this further. We do expect SSL pup adiposity prior to the first fast to vary as a broad range of perinatal period durations (Table 1) have been observed, ranging from 3.2 days (Año Nuevo, CA in 1973; Hood and Ono, 1997) to 17 days (Medny Island, Russia in 2007; Burkanov *et al.*, 2011). Pup suckling efficiency and stomach volume increase as a pup ages, placing upper limits upon the quantity of milk ingested (Higgins *et al.*, 1988). Female Antarctic fur seal (*Arctocephalus gazella*) pups lost mass at a significantly greater rate than male pups (−2.55% and −2.12%, respectively) during fasting periods; male pups also had significantly higher growth rates, suggesting that at this age, sexual-dimorphism may account for mass-specific rates of decline in body mass during fasting periods (Guinet *et al.*, 1999). Collectively, the variability in these pup characteristics may result in differences in the onset of fasting Phases *II and III*.

### Biological significance of pup sex differences

The difference noted in fasting duration between sexes was not unexpected for a sexually-dimorphic, polygynous species. Parental investment theory suggests that the increased investment in male offspring is a means to maximizing lifetime fitness (Trillmich, 1996). Consistent with that theory, we found that the presence of female pups was significantly greater in *Long* fasts than male pups, irrespective of subpopulation, suggesting that the energy reserves of a greater proportion of male pups were adequate to sustain the protein conservation phase for the duration of the dam’s absence. Male pups of sexually-dimorphic pinnipeds tended to be born larger than females of the same species (within same geographical region), the sex ratio of pups was more-commonly skewed towards males, male pups had a faster growth rates and male pups consumed greater absolute milk volumes (before mass-correction) than female pups (Kerley, 1985; Anderson and Fedak, 1987; Oftedal *et al.*, 1987; Trillmich, 1990; Lunn *et al.*, 1993; Ono and Boness, 1996; Boltnev and York, 2001; Winship *et al.*, 2001; Bradsaw *et al.*, 2003). New Zealand fur seal (*Arctocephalus forsteri*) dams had significantly longer foraging trips when rearing male pups than females (Goldsworthy, 2006). Anderson and Fedak (1987) further calculated that the energetic investment in rearing male grey seal (*Halichoerus grypus*) pups was 10% greater than that of females for the combined gestation and lactation periods.

The sex-ratio trend typical of sexually-dimorphic pinnipeds held true for 10 of 12 SSL subpopulations in our study. The largest deviation from this trend was observed in pups in the western Aleutian Islands, an area where population abundance has not stabilized and continues to decline and where elevated mercury concentrations have been repeatedly documented in young pups, as previously discussed. A female-skewed sex ratio could signal that the extra costs associated with rearing male pups were significant for some SSL dams. Our evaluation of this beyond speculation is limited as 1) the sex ratio of pups we provided was based off of a subsample of the pups born into a subpopulation, and 2) handling events aimed to occur after the peak of the pupping period, but before the pupping period commences such that additional pups were likely born post-handling. The plasticity of these sex-specific differences in maternal investment with respect to environmental conditions is not well understood for SSL. We suspect that with increased and more balanced sample sizes among subpopulations, a more distinct pattern may emerge.

## Conclusions

Plasma analyses used to characterize fasting phase provide valuable insight into the variability in maternal attendance at the subpopulation level. Where archived plasma samples exist, the distribution of fasting phases for a snapshot in time can be created retrospectively where traditional maternal attendance studies were not conducted. We suggest this application of plasma metabolite comparisons, used in combination with other health assessment metrics, is a valuable long-term monitoring tool for examining variability in fasting duration and condition in pups at a given rookery.

While results for the majority of pups from most subpopulations in this study are not a cause for concern, this study highlights three subpopulations where further investigations into maternal attendance and resultant pup condition are warranted. These three subpopulations have had limited to no prior maternal attendance studies conducted: none conducted in the western Aleutian Islands, Seguam Island in the central Aleutian Islands was studied in 1997 and rookeries in southern Southeast Alaska were monitored from 1992 to 1997 (Table 1). Due to vast differences in population growth within the western and central Aleutian Islands and the southern portion of Southeast Alaska, we suspect the elevated proportions of *Long* fasts in these areas are influenced by different drivers. Conspecific competition should be considered as a leading contributing factor in Southeast Alaska, while a density independent factor(s), such as mercury exposure, is more likely within the western and central Aleutian Islands. Broader scale maternal attendance studies at these locations would provide additional information regarding timing of the parturition period at the rookery, perinatal period duration, fasting duration and shore visit frequency and durations, and may aid interpretation of our findings. Although our interpretation of causes of longer fasts is limited from available data on the potential explanatory factors discussed herein, potential conservation implications could result from fasting for extended periods, compromising lean body mass and impeding allocation to somatic growth, development and lipid storage by pups.

## Supplementary Material

Web_Material_coad084

## Data Availability

Data from this project are publicly available through the dryad data repository website: https://doi.org/10.5061/dryad.66t1g1k76.
