# Supplementary material for: Fasting durations of Steller sea lion pups vary among subpopulations—evidence from two plasma metabolites
Source: Conserv Physiol. 2023 Nov 24;11(1):coad084. doi: 10.1093/conphys/coad084 (PMC10673819; doi:10.1093/conphys/coad084)
Supplement: Web_Material_coad084 [file web_material_coad084.pdf]

SUPPLEMENTAL DATA

Comparison Plasma Metabolites in Chemically- and Physically-Restrained SSL Pups

Purpose

This analysis was conducted to evaluate the potential that the capture stress imposed upon SSL pups during physical restraint may result in increased concentrations of plasma [BUN] (e.g., capture stress mediated myopathy) and plasma [ß-HBA] (e.g., glucocorticoid driven lipolysis) prior to blood sampling (Harcourt *et al.*, 2010; Champagne *et al.*, 2012; Atkinson *et al.*, 2018; Breed *et al.*, 2019; Dinesh *et al.*, 2020). This is important to evaluate as, if physical capture stress were significant, it may inflate the proportion of pups classified into the *Long* fasting group, biasing our interpretation of overall results.

Prior Studies

During the first year of their study, Myers *et al*. (2010) found that SSL pups born in southwest Alaska had significantly lower [cortisol] than those born in eastern Russia or Southeast Alaska. However, in the following year, no regional differences were identified, but male pups had significantly lower [cortisol] than female pups (Myers *et al*., 2010). Keogh et al. (2013) measured serum cortisol in 61 young SSL pups from the same eastern Gulf of Alaska rookery (Chiswell Island) captured in 2005, 2007, and 2008. Pups were chemically restrained in 2005 and 2008, and physically restrained in 2007. [Cortisol] were significantly higher in 2008 than the other two years, therefore not attributable to capture technique. The authors suspect the interannual difference in [cortisol] were influenced by annual differences in sample size and variability in sample collections times among years. A regression of [cortisol] and the elapsed time to blood collection (between researchers' arrival on the rookery and sampling) followed a weak negative relationship (r^2=0.220). No differences in [cortisol] were found between sexes, known age (range 5-40 days), body condition index, or other hormones measured (aldosterone, leptin, T_4, T_3). The observed [cortisol] in this study were considered to be in response to the initial disturbance to the rookery.

Selection of Subset of Subpopulation Evaluated

Captures at rookeries on Ugamak Island (eastern Aleutian Islands subpopulation) provided a unique opportunity to control for other potentially confounding variables. The eastern Aleutian Islands is a subpopulation that has had modest population recovery in comparison to adjacent subpopulations (central and western Aleutian Islands), therefore removing confounding factors related to population decline. While both capture techniques were applied on Ugamak Island SSL pups, we are unable to control for variables associated with interannual variability as pups were physically restrained during sample years 1991, 1993, and 1995–1997 and chemically restrained during 2003, 2005, and 2009. The decision (and subsequent permitting and approvals) to physically or chemically restrain pups was based upon the types of procedures and length of handling required for procedures on pups in those respective years. Pups in all years were subject to the disturbance of researcher presence on the rookery and physical handling for mass measurement and transfer to the sampling station. Lastly, the sampling regime for Ugamak Island provided appropriate sample sizes for this comparison. Quantitative analyses were conducted using open-source software R (R Core Team, 2023). Statistical differences attributed to restraint technique were evaluated using the Kruskal-Wallis non-parametric test.

Results

The relative concentrations of plasma [BUN] and [ß -HBA] (Supplementary Figure 1) suggest no differences due to restraint technique. The percent distribution of pups into fasting categories was consistent with findings for the eastern Aleutian subpopulation (Supplemental Table 1, Figure 6). Our statistical tests found no difference in plasma [BUN] (Kruskal-Wallis X^2=0.5624, df=1, p=0.4533) or plasma [ß -HBA] (Kruskal-Wallis X^2=0.3466, df=1, p=0.5560) with respect to restraint technique (Supplemental Figure 2).

Conclusions

Based upon this review of a subset of study animals from one subpopulation, we find no evidence to justify separating SSL pups restrained by chemical and physical techniques into separate groups for the purposes of evaluating fasting category among subpopulations.

References

Atkinson S, Dieraug LA (2018) Stress and Marine Mammals. In: Gulland FMD, Dierauf LA, Whitman KL, eds. CRC Handbook of Marine Mammal Medicine, Third Edition. CRC Press, Boca Raton, FL, USA, pp 153–168.

Breed D, Meyer CRM, Steyl JCA, Goddard A, Burroughs, R, KohnTA (2019) Conserving wildlife in a changing world: Understanding capture myopathy–a malignant outcome of stress during capture and translocation. *Conserv Physiol* 7 (1): coz027; doi:10.1093/conphys/coz027.

Champagne CD, Houser DS, Costa DP, Crocker DE (2012) The effects of handling and anesthetic agents on the stress response and carbohydrate metabolism in northern elephant seals. *PLoS ONE* 7(5): e38442. https://doi.org/10.1371/journal.pone.0038442.

Dinesh M, Thakor JC, Yadav HS, Manikandan R, Anbazhagan S, Kalaiselvan E, Pradeep R, Khillare RS, Sahoo M (2020) Capture myopathy: An important non-infectious disease of wild animals. *Int J Curr Microbiol App Sci* 9(4):952-962.

Harcourt RG, Turner E, Hall A, Waas JR, Hindell M (2010) Effects of capture stress on free-ranging reproductively active male Weddell seals. *J Compar Physiol A* 196:147-154.

Keogh MJ, Atkinson S, Maniscalco JM (2013) Body condition and endocrine profiles of Steller sea lion (*Eumetopias jubatus*) pups during the early postnatal period. *Gen Comp Endocrinol* 184:42–50.

Myers MJ, Litz B, Atkinson S (2010) The effects of age, sex, season and geographic region on circulating serum cortisol concentrations in threatened and endangered sea lions (*Eumetopias jubatus*). *Gen Comp Endocr* 165(1):72–77.

R Core Team (2023) R: A language and environment for statistical computing. R Foundation for Statistical Computing, Vienna, Austria.

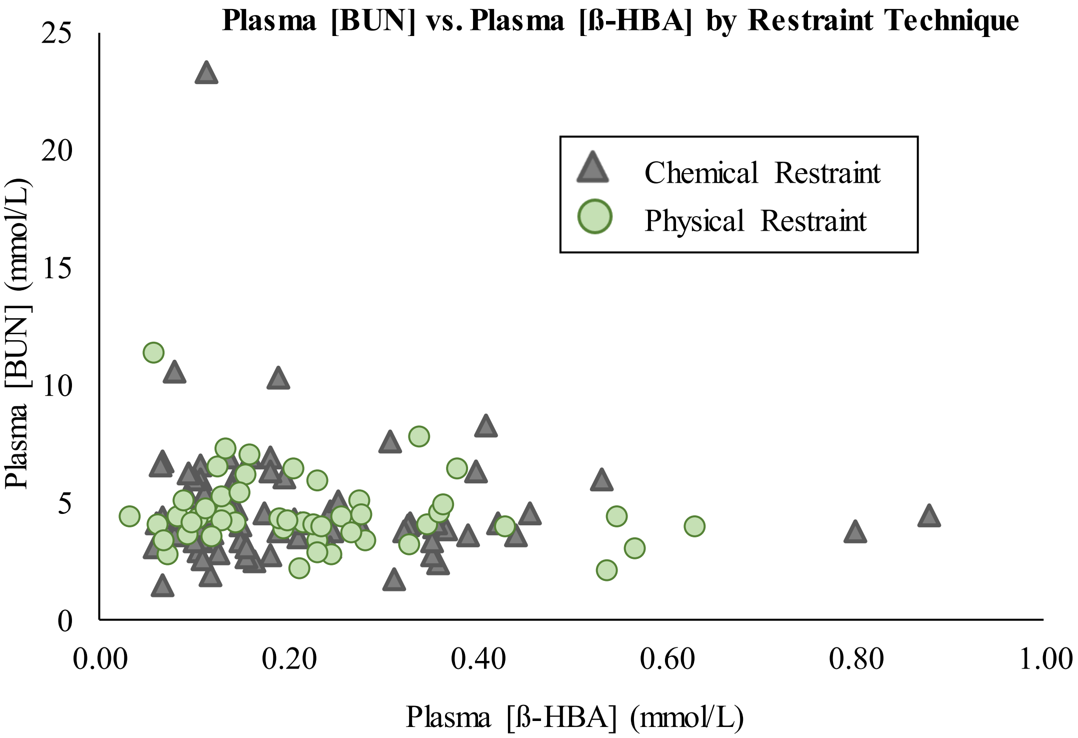

Supplemental Figure 1 Relative concentrations of plasma blood urea nitrogen ([BUN]) and plasma ß-hydroxybutyrate ([ß-HBA]) for SSL pups restrained by chemical (n=78, grey triangles) and physical (n=50, green circles) techniques at Ugamak Island.

Concentration of Plasma Metabolites by Capture Technique

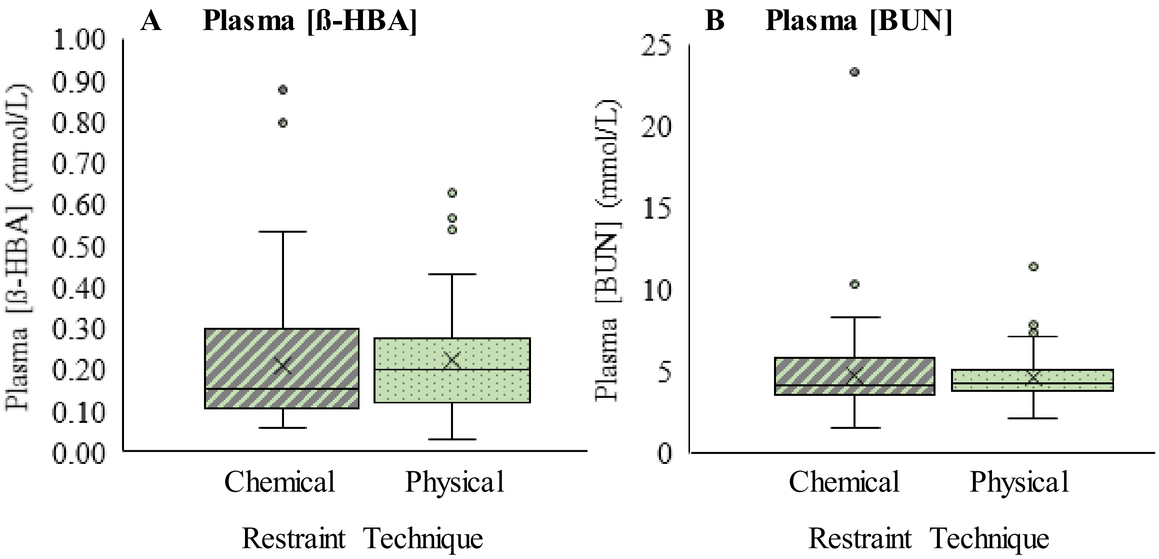

Supplemental Figure 2 (A) Plasma ß-hydroxybutyrate ([ß-HBA]) and (B) plasma blood urea nitrogen ([BUN]) for SSL pups restrained by chemical (n=78, striped bars) and physical (n=50, dotted bars) techniques at Ugamak Island. No statistically significant differences were identified between techniques for either metabolite (Kruskal-Wallis tests, $p>0.45$).

Supplemental Table 1 Comparison of the fasting category assignments of SSL pups restrained by chemical and physical techniques. The number of pups (n), percent of total pups in fasting category (%), the mean plasma blood urea nitrogen (BUN), and the mean plasma ß-hydroxybutyrate (ß-HBA) concentrations in each fasting category are provided for each restraint technique. Metabolite concentration thresholds for each fasting category can be found in Table 3.

Restraint Technique	Fasting Category	n	%	BUN (mmol/L)	ß-HBA (mmol/L)
CHEMICAL	Fed / I	55	70.5%	4.39	0.13
	II	14	17.9%	3.54	0.39
	II-III	6	7.7%	6.22	0.50
	III	3	3.8%	14.75	0.13
	All Pups	78	100%		
PHYSICAL	Fed / I	38	76.0%	4.48	0.16
	II	6	12.0%	3.43	0.47
	II-III	5	10.0%	5.68	0.40
	III	1	2.0%	11.38	0.06
	All Pups	50	100%		
